# Supplementary material for: Nutritional intakes of highly trained adolescent swimmers before, during, and after a national lockdown in the COVID-19 pandemic
Source: PLoS One. 2022 Apr 5;17(4):e0266238. doi: 10.1371/journal.pone.0266238 (PMC8982883; doi:10.1371/journal.pone.0266238)
Supplement: Attachment — Submitted filename: Response to Reviewers.docx [file pone.0266238.s002.docx]

**Journal Requirements**

1. Please ensure that your manuscript meets PLOS ONE's style requirements, including those for file naming. The PLOS ONE style templates can be found at 
[https://journals.plos.org/plosone/s/file?id=wjVg/PLOSOne_formatting_sample_main_body.pdf](https://eur02.safelinks.protection.outlook.com/?url=https%3A%2F%2Fjournals.plos.org%2Fplosone%2Fs%2Ffile%3Fid%3DwjVg%2FPLOSOne_formatting_sample_main_body.pdf&data=04%7C01%7CJosh.Newbury%40mail.bcu.ac.uk%7Ce5b37526cdb046c477fd08d9df4895a3%7C7e2be055828a4523b5e5b77ad9939785%7C0%7C0%7C637786325103708067%7CUnknown%7CTWFpbGZsb3d8eyJWIjoiMC4wLjAwMDAiLCJQIjoiV2luMzIiLCJBTiI6Ik1haWwiLCJXVCI6Mn0%3D%7C3000&sdata=MVqyYZ9tRxlCl3K5bszfBeuHANv6AwHDwMeydKfwCdI%3D&reserved=0) and 
[https://journals.plos.org/plosone/s/file?id=ba62/PLOSOne_formatting_sample_title_authors_affiliations.pdf](https://eur02.safelinks.protection.outlook.com/?url=https%3A%2F%2Fjournals.plos.org%2Fplosone%2Fs%2Ffile%3Fid%3Dba62%2FPLOSOne_formatting_sample_title_authors_affiliations.pdf&data=04%7C01%7CJosh.Newbury%40mail.bcu.ac.uk%7Ce5b37526cdb046c477fd08d9df4895a3%7C7e2be055828a4523b5e5b77ad9939785%7C0%7C0%7C637786325103708067%7CUnknown%7CTWFpbGZsb3d8eyJWIjoiMC4wLjAwMDAiLCJQIjoiV2luMzIiLCJBTiI6Ik1haWwiLCJXVCI6Mn0%3D%7C3000&sdata=uFK21ovE0QYCGa4%2FqQMYutKCsbmta0ms%2F08HFUb2tfQ%3D&reserved=0)

- Thank you, we have adhered to these requirements throughout the manuscript.

2. Please note that in order to use the direct billing option the corresponding author must be affiliated with the chosen institute. Please either amend your manuscript to change the affiliation or corresponding author, or email us at [plosone@plos.org](mailto:plosone@plos.org) with a request to remove this option.

- The corresponding author is S. Andy Sparks of Edge Hill University, who has an affiliation with PLoS one.

3. Please include captions for your Supporting Information files at the end of your manuscript, and update any in-text citations to match accordingly. Please see our Supporting Information guidelines for more information: [http://journals.plos.org/plosone/s/supporting-information](https://eur02.safelinks.protection.outlook.com/?url=http%3A%2F%2Fjournals.plos.org%2Fplosone%2Fs%2Fsupporting-information&data=04%7C01%7CJosh.Newbury%40mail.bcu.ac.uk%7Ce5b37526cdb046c477fd08d9df4895a3%7C7e2be055828a4523b5e5b77ad9939785%7C0%7C0%7C637786325103708067%7CUnknown%7CTWFpbGZsb3d8eyJWIjoiMC4wLjAwMDAiLCJQIjoiV2luMzIiLCJBTiI6Ik1haWwiLCJXVCI6Mn0%3D%7C3000&sdata=ZAFdq68DpvkJGPFOkK%2BKFMIa9oVXNwbZLRwS2MOwyjE%3D&reserved=0).

- We have added the following caption ‘**S1 Data.** Anonymised data set’ after the reference section (line 630).

**Reviewer’s Comments**

Reviewer #1: In this study, the authors analyse the nutritional intakes of high-performance adolescent swimmers before, during and after lockdown. Using a longitudinal assessment of self-reported dietary intakes, they study the effect of lockdown restrictions on dietary practices of young swimmers benefiting from a virtual nutrition support. Based on this finding, they reported that adolescents swimmer seems to be able to appropriately adjusted their energy and macronutrient intakes to match their changing energy demands. The authors suggest promoting and studying the relevance of virtual nutrition support for athletes, especially in case of further periods of social isolation. Some important considerations are highlighted in this paper, but the importance of nutrition and the impact of the results found on the health and performance of young athletes should be more highlighted.

- We would like to thank the reviewer for the constructive comments, which we believe has helped to produce a stronger manuscript. We have attempted to satisfy all of the comments below and feel that we now have more discussion of the impacts of nutrition of health/performance outcomes. However, since we did not collect any performance/health measures, the paper is still mostly focused on the descriptive changes that occurred. We are more than happy to make further amendments at your discretion.

  Introduction

Line 77 – The reference 17 is prior to COVID and concerns restrictive practices, the quote should not be at the end of the sentence to avoid confusion.

- To avoid confusion and to temper the statement suggesting that athletes may have taken up restrictive diets due to COVID, lines 77-79 have been rearranged within the manuscript to state: “Based on this notion, it is likely that some adolescent swimmers turned to contemporary low-energy diets (e.g., low-fat, ketogenic, intermittent fasting [18]) in order to preserve body composition when physical activity was reduced”.

Line 81 – What is the reference for this value for 1,5g/kg/d? not the same in the paper from Shaw nor Desbrow.

- This protein value was based on the example nutrition tables of Shaw et al., (2014), who recommend swimmers ingest 1.5-1.8 g/kg/d for high volume training weeks, and 1.7-2.0 g/kg/d for high intensity training weeks. Desbrow et al., (2014) were not referenced for this value however, their recommendation does suggest that “the most prudent approach for competitive adolescent athletes is to follow the guidelines for adult athletic populations regarding protein consumption”. The specific value of <1.5 g/kg/d being suboptimal was in reference to Jukic et al., (2020), who recommend increasing protein intake during lockdown (i.e., consuming less than the minimal recommended by Shaw et al., (2014) during lockdown would be suboptimal). Admittedly, the in-text recommendation of Shaw et al., (2014) further breaks down protein intake to suggest 4-5 x 0.3 g/kg equally spaced protein meals/snacks per day, although including this may add further confusion since meal frequency was not included in this study. On reflection, the references within this sentence did feel out of place, and have been rearranged in lines 82-84 to state: “The combination of these factors could also result in the occurrence of suboptimal protein (<1.5-1.7 g∙kg BM∙day^-1^ [7]) and micronutrient intakes [21] being consumed, which is a further cause for concern as these can increase the rate of muscle atrophy [9] and lead to deficiencies [22], respectively.” We hope this is clearer to the reader, although we are open to further suggestion.

Dietary references (for energy/CHO/fat) and RNI as part of the results and discussion should appear in the introduction.

Line 215 – carbohydrate without -s. I even think it would be good to harmonize the rest of the manuscript with CHO.

- These RNIs have been added to lines 67-68 in the introduction in the amended sentence: “Large reductions in training hours are also likely to decrease daily energy expenditure (DEE), which could result in body fat accretion if the recommended dietary practices for swimmers (i.e., energy: 35-75 kcal∙kg BM∙day^-1^ [7], carbohydrate (CHO): 3-8 g∙kg BM∙day^-1^ [7], fat: 1-2 g∙kg BM∙day^-1^ [10]) are not reduced to meet the new training demands [11]”. This has also introduced the term ‘carbohydrate’, which has been harmonized for the remainder of the manuscript.

Methods
What are your eligibility criteria? How did you determine the sample size? with a population of 13 athletes, I think you would have to justify a sample size at entry or a power test at exit.

- This study retrospectively analysed food diary data that was collected as part of a nutrition service for a high-performance swimming club, therefore the eligibility criteria were that all swimmers were nationally competitive within their age groups. During lockdown, some swimmers submitted unsatisfactory contributions (i.e., two-day food dairies, nutrition recalls, no submissions), which were accepted as these data were not originally collected for research purposes. Unfortunately, this meant that these individuals had to be excluded from statistical analyses, which along with other dropouts, resulted in this study utilising only half (13/26) of the potential participants. This information has been included in the first paragraph of the methods to make our sample of research constraints clearer to the reader (Lakens, 2021).

Explain what sport science support is. The nutritional support discussed afterwards is not clearly defined

- We agree that ‘nutrition support’ was loosely discussed, despite likely having a large influence on the resultant outcomes. A ‘nutrition support’ section (175-194) has therefore been added to the methods section to provide more clarity on the kind of support the swimmers were receiving at this time. Pre-lockdown, this involved monthly one-to-one consultations (dependent of engagement) and weekly group education sessions (varying topics) with an undergraduate nutrition student. During lockdown, lots of resources (i.e., PDF files) were made for swimmers and parents to support immunity, muscle retention, exercising from home, and weight maintenance. The swimmers were granted weekly one-to-one consultation support, but this was met with varying levels of engagement (i.e., some daily, some monthly). Scheduled group activities were then introduced online, whereby the undergraduate student provided a cooking workshop and a nutrition quiz each week. After lockdown, the support was mostly resource (PDF) based since social interactions were limited, with a few swimmers retaining nutrition consultation. Based on the unforeseen nature of this study, detailed records of education sessions, interventions, attendance at group sessions, or engagement with one-to-one support were not collected.

Explain that the nutritional values will be reported in absolute (EI, macro, minerals, vitamins) and relative to body weight (EI, macro, caffeine). Maybe even specify the reason?

- An explanation has been added for how and why measures were reported with the statement: “all nutrients were reported in absolute values, with energy, macronutrients, and caffeine further discussed as relative values to standardise intakes based on body mass” (lines 132-134).

Anthropometric Data
Specify the conditions of weighing (fasting, in underwear?) and the instructions given for home weighing.

- The following statements have been added to clarify weighting conditions: pre- and post-lockdown – “all measurements were collected before morning training (4:45 AM) whilst the participants were in swimming costumes” (lines 149-150), during lockdown – “in an attempt to standardise measures, these were requested after breakfast whilst wearing underwear” (lines 151-152).

Training Information
We don't know how you got the training characteristics data (activity diary filled out by the athletes?)

- Swimming volume was attained via communication with the head swimming coach, who logged all swimming training sessions for the club (and self-reported sessions during lockdown). Exercise records were kept by the swimmers and sent to coaches to facilitate training during lockdown, which was used to calculate total training time. Pre- and post-lockdown, the swimmers were asked to detail any additional exercise outside of swimming training, which was added to total training time. This information has been included to the training characteristics section in lines 161-162, and 170-173).

Statistical Analysis

For more fluidity in reading, separate the statistical analyses of the pilot study from other analyses.
Clarify what is done if normality is not respected?

- We have now separated the main statistical analyses from the pilot study to improve the fluidity in reading. No violations of normality were observed, but a non-parametric Welch test would have been conducted if necessary.

  Anthropometric Responses
  I don't know if you have the data to calculate it ... but the biological gae estimated from the sitting height for example would be an interesting element to discuss the homogeneity of the group.
- This would have been a great insight into the biological changes that occurred over the lockdown, however, we only have sitting height data from October 2019. We will certainly consider using this measurement in future research.

  Energy and Macronutrient Intakes
  l.218 Can we really say that there are even small differences with such significance? If all the subjects had the same tendency, argue in this sense. Especially since in the discussion it is specified that “After the lockdown, the swimmers consumed a diet that was no different in energy, macronutrient, or micronutrient content compared to before COVID-19”.
- Upon further review of the individual results, we identified that 8 participants increased, 4 maintained, and 1 reduced fat intake from during to after lockdown. In comparison to protein (11 increased, 2 decreased) and CHO (13 increased, 0 decreased), this variability likely contributed towards the lack of statistical significance. The view that fat intakes were unchanged will therefore be tempered for the discussion of results (lines 243-247).

l.224 Quote here the reference 7 (I did not find on which reference Shaw et al bases itself to give this value?)

- These values were found in the Tables 1b and 1c of Shaw et al. (2014), which give recommendations based on example high volume (energy: 40-75 kcal/kg/d, CHO: 3-7 g/kg/d, protein: 1.5-1.8 g/kg/d) and moderate volume training weeks (energy: 35-55 kcal/kg/d, CHO: 3-8 g/kg/d, protein: 1.7-2.0 g/kg/d). These values are somewhat similar to the Stellingwerf et al. (2011) recommendations for power athletes (including swimmers) in general prep (energy: 50-70 kcal/kg/d; CHO: 6-12 g/kg/d, protein: 1.5-1.7 g/kg/d) and specific prep phases (energy: 40-65 kcal/kg/d; CHO: 6-10 g/kg/d; protein: 1.5-1.7 g/kg/d), therefore their recommendations for relative fat (general prep: 1.5-2 g/kg/d; specific prep: 1-1.5 g/kg/d) were deemed appropriate.

l.228 Do they consume more or less CHO than recommended?
l.231 It would be better to have a reference for excess and insufficient amount of protein intake
l.236 Reference for insufficient amount of fat intake

- We have rewritten this section (lines 258-269) to focus more on the individual variances that occurred around the recommended intakes, rather than stating language such as ‘insufficient’ and ‘excess’. References for the recommended intakes have now been introduced as per your previous comment, which should now mean that the readers can relate back to this information and improve readability. Upon review, we have also reduced the word count of this section since most of these observations can be made from Fig 1. We have therefore focused on the most notable data, whereby CHO was not further discussed beyond an increased CV. We hope that these changes make analysis of this individual data more fluid, but we are open to further suggestion.

Tables 2, 3 and 4
Specify that the data are means and standard deviation
Specify that a = different to before (January) and b = different to during (April)

- These have now been amended for all tables (lines 107, 255-256, 297-298, 316-318, and 352-354).

Carbohydrate and Lipid Components

l.253 participants demonstrated an altered amount of fiber, and monounsaturated fat compared to what? do they respect the recommendations

- This sentence was meant to highlight that changes occurred between measuring time points (i.e., before vs. after lockdown). We have therefore amended lines 277-278 to state “changes in dietary fibre (F=13.4, *p*<0.001, Pη^2^=0.53), sugar (F=12.6, *p*<0.001, Pη^2^=0.51), and monounsaturated fat (F=5.8, *p*=0.009, Pη^2^=0.33) intakes were identified over the study timeframe”. Whether they respected the recommendations was not previously discussed, however, we have now compared fibre (line 279) and sugar intakes (lines 284-286) to the RNIs. No data was available for monounsaturated fats within the RNIs to compare.

l. 294 put the reference (30)

- This sentence has now been removed, and RNIs have now been added to Tables 4 and 5 (lines 314-318, and 350-354).

l.299 the 13 before and 3 after swimmers are all female?

- This was a reference to individual swimmers, i.e., participant 1 and participant 13 were not achieving energy, calcium, or iron recommendations. We have now removed this to avoid confusion.

  Discussion
  l. 336 and 337 “this was the first study” twice, it's a bit repetitive
- This has been amended in lines 362-364.

l.343 to specify perhaps that the NRIs are not specific to the young athletes and quoted the citation reference

- Thank you for your suggestion. We have specified that the RNIs were for British adolescents, but continued to state “which is concerning as athletes may have greater micronutrient needs” (lines 367-369), which we have now mentioned later in the discussion.

l.347 the difference in size between before and after is about 3 cm, right? (1.66 ± 0.09 vs. 1.69 ± 0.08 m; p = 0.002, g = 0.27).

l.363 or +3cm?

- This is correct, this was my mistake and has been amended throughout the discussion.

Can we say that without having evaluated the impact of the nutritional program on this group of swimmers? the ability of some young swimmers to modify their diet to meet their needs may be enhanced by the nutrition program (hence the importance of explaining the objectives of the program beforehand)

- This is very true, and we have therefore tempered this statement within the manuscript by mentioning that the swimmers were receiving nutritional support (lines 372-374).

It would be interesting to discuss the gender effect. Indeed, as Shaw et al. (2014) point out, males are more likely to match their food intake to their expenditure (at least in the sense of covering their needs when they increase).

- We agree that this is an interesting point, and have attempted to include this as a discussion point in lines 428-432.

l.379 RNI 15-18 (l.343 RNI for adolescents aged 11-19years) Is this normal or a mistake?

- The British RNI have recommendations for 11-19 years (recently updated to 11-16 years) for some nutrients (i.e., fibre), and recommendations for 11-14/15-18 years for vitamins and minerals. To account for both categories, we originally stated the 11-19 years range, although this was a mistake since the main discussion points were on vitamins and minerals (and there are slight differences in 11-14 vs. 15-18 years recommendations). This mistake has been amended in line 426.

l.387 Add a reference

- We have included a reference and statement to suggest that micronutrient intakes may not be reliable, since these values have not been measured in the validation study (i.e., Costello et al., 2017) (lines 433-434).

l.396 Perhaps it would be necessary to moderate this information by adding "to our knowledge".

- We were unsure which statement this pertained to however, we have undergone large amendments of the discussion section which we hope has resolved such issues.

l.414 and in boys? if this is the case, it is possible to introduce the REDS ?

- We have reduced our discussion point surrounding female athlete triad and RED-S since we had no measure of energy balance. We have included this lack of measure, and therefore inability to calculate energy deficiency as a limitation, rather than further discussing what we did not measure (lines 438-444).

l.417 the paragraph on the method of assessing food intakes is either a methodological discussion point and should be at the beginning of the discussion or a limitation, to be discussed as such

- We have removed this as a limitation since we had already introduced this issue and taken measures to counteract this within the methods section.

What is the impact of variations in food intake on the health/performance of young athletes? Among other things, when they no longer respect the recommendations? Does the significant difference have a clinical meaning? To emphasize the contribution of these results to the research in nutrition of athletes.

- We agree that we have perhaps not provided much practical application to our findings. The reason we have done this is that as we are aware of the already lengthy manuscript, so are cautious of providing too much explanation beyond what we actually investigated (changes in dietary intake). We have mentioned that no performance or health measures were taken as a limitation (lines 447-451). Doing this would have enabled more confident statements on health-related practical applications, but in the circumstances of the data collection, this was not possible.

Conclusion
l.439 the previous studies on swimmers were not done under the same conditions (covid ...) quote the studies on other sports but done during the covid or specify the conditions?

- We have now removed this comparison to other studies and specified that our data was found in sport nutrition supported swimmers (line 456).

l.439 There is a significant reduction in training (in hours and km/week) and a significant reduction in food intake but are these two reductions of the same order? is it possible to say that the athletes have approprietly adjuted their energy and macronutrient intakes?

- We have removed this comparison from the conclusion.
